# Development of a Biodegradable Polysaccharide‐Based Polymer Film From *Hibiscus sabdariffa* Sepal Mucilage and Carboxymethyl Cellulose

**DOI:** 10.1002/fsn3.72335

**Published:** 2026-09-09

**Authors:** Maryam Beigomi, Mohammad Amin Mashhadi, Moharram Valizadeh, Farzaneh Farajian Mashhadi

**Affiliations:** ^1^ Department of Food Industry, School of Medicine Zahedan University of Medical Sciences Zahedan Iran; ^2^ Health Promotion Research Center Zahedan University of Medical Sciences Zahedan Iran; ^3^ Food and Drug Deputy Zahedan University of Medical Sciences Zahedan Iran; ^4^ Department of Physical Geography University of Sistan and Baluchestan Zahedan Iran; ^5^ Department of Pharmacology, School of Medicine Zahedan University of Medical Sciences Zahedan Iran; ^6^ Pharmacology Research Center Zahedan University of Medical Sciences Zahedan Iran

**Keywords:** biodegradable, carboxymethyl cellulose, *Hibiscus sabdariffa* sepal mucilage, polysaccharide‐based polymer film

## Abstract

This research investigated the feasibility of utilizing *Hibiscus sabdariffa* sepal mucilage (HSSM) as a novel biopolymer matrix in combination with carboxymethyl cellulose (1%, w/w) and varying glycerol content (10%, 15% and 20%, w/w) for the development of biodegradable films. The microstructural, thermal, mechanical, and physical properties, as well as the antioxidant and antimicrobial potential, of the resulting biodegradable films were evaluated. Results indicated that increasing glycerol content led to significant increases in film thickness, moisture content, solubility, water vapor permeability, and elongation at break (EB%), confirming its plasticizing effect within the HSSM‐based matrix. Conversely, tensile strength, melting temperature, and glass transition temperature (Tg) decreased significantly with increasing glycerol content. Color analysis showed that the films remained transparent, with slight greenish or reddish coloration at higher glycerol levels. Scanning electron microscopy revealed that glycerol‐containing films exhibited a more uniform and homogeneous morphology without visible cavities or fractures compared to films without glycerol. Furthermore, the unplasticized HSSM film demonstrated notable antioxidant activity, and the optimized HSSM‐CMC film (10% glycerol) exhibited significant antimicrobial activity against *Staphylococcus aureus* and *Bacillus cereus* . These findings suggest that HSSM‐CMC films have strong potential for active food packaging applications. Overall, combining HSSM with carboxymethyl cellulose led to the development of biodegradable films with improved structural integrity and desirable functional attributes.

## Graphical abstract

A biodegradable HSSM–CMC composite film was developed from *Hibiscus sabdariffa* sepal mucilage (HSSM, 1.25%) and carboxymethyl cellulose (CMC, 1%), using glycerol at different concentrations (10%–20%) as a plasticizer. The resulting films were evaluated for their mechanical, barrier, thermal, antioxidant, and antimicrobial properties. The developed HSSM–CMC films demonstrate potential for application as active biodegradable food packaging materials.

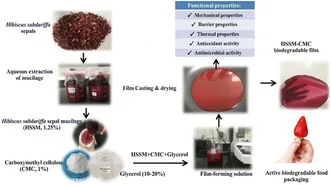

## 1 Introduction

Packaging materials play a crucial role in protecting products during storage, transport, and delivery. Global plastic production and consumption have been increasing due to economic expansion and population growth. Synthetic polymer‐based packaging materials are non‐biodegradable, and their widespread use has resulted in contamination and environmental problems. Chemical monomers released from plastics under heat and sunlight may also pose health risks. Packaging accounts for a large portion of plastic use, and nearly two‐thirds of packaging waste originates from this sector (Lenz 2005; Mostafavi and Zaeim 2020). Several attempts have been made to develop biodegradable materials as sustainable alternatives. Natural polymers from renewable sources are considered promising substitutes for synthetic polymers. Biopolymer films are biodegradable and can be decomposed by microorganisms into simple components (Ramadan et al. 2025). Such films exhibit favorable mechanical strength and barrier properties, including resistance to gases, moisture, and water vapor (Hasan et al. 2020). Biopolymer films can be categorized as polysaccharide, protein, lipid, or composite types. Polysaccharide films, including starch derivatives, dextrins, cellulose derivatives (such as carboxymethyl cellulose), gums, and mucilages, are among the most important. Their hydrophilic nature allows water absorption while effectively blocking lipids and odor molecules. These coatings reduce oxygen transport and provide stability in oxidation‐sensitive systems. Although natural polymers cannot fully replace synthetic ones, they offer a cost‐effective and eco‐friendly alternative for developing sustainable biodegradable polymer systems (García et al. 2016).

Among natural polysaccharide sources, seed and plant mucilages, including those obtained from chia, basil, quince, flaxseed, and okra seeds, have been extensively investigated for the development of biodegradable and edible packaging films, owing to their favorable film‐forming, gelling, and antioxidant properties (Dick et al. 2015). Biopolymer‐based packaging materials, in general, still exhibit limitations related to mechanical performance, moisture sensitivity, and limited functional (antimicrobial and antioxidant) activity, which restrict their practical application as viable alternatives to synthetic plastics (Khazaei et al. 2014). In particular, single‐component mucilage films may require plasticizers to improve flexibility and prevent brittleness, which may reduce tensile strength and increase water vapor permeability, as reported for basil, chia seed, and quince seed mucilage films (Dick et al. 2015; Khazaei et al. 2014; Jouki, Yazdi, et al. 2013). These limitations have led to increasing interest in composite packaging systems in which different biopolymers are combined to improve mechanical, barrier, and functional properties. Recent advances in biopolymer‐ and PBAT‐based composite packaging, including nanohybrid systems and polyphenol‐crosslinked cellulose/chitosan films (Hou et al. 2025; Venkatesan, Mayakrishnan, et al. 2025; Venkatesan, Kamaraj, et al. 2025), have been investigated for improving barrier, mechanical, and antimicrobial performance to overcome some of these limitations.

Despite the growing interest in plant‐derived mucilages, *Hibiscus sabdariffa* sepal mucilage (HSSM), an underexplored plant resource, has received relatively limited attention as a film‐forming biopolymer, particularly in combination with structural polysaccharides such as carboxymethyl cellulose (CMC) for enhancing mechanical and barrier properties. *Hibiscus sabdariffa*, a member of the Malvaceae family, produces sepals that contain mucilage and bioactive compounds, including citric acid, vitamin C, anthocyanins, and flavonoids, which contribute to antioxidant and functional properties (Bahrami Feridoni and Khademi Shurmasti 2020). The mucilage, a high‐molecular‐weight carbohydrate, is soluble in water, resistant to low pH, and valued for its stability and emulsification properties. Due to these characteristics, HSSM is considered a promising biopolymer material for producing biodegradable polymer films. However, despite these promising characteristics, its direct application as the primary film‐forming matrix remains largely unexplored. Although *Hibiscus sabdariffa* ‐derived compounds, such as extracts and isolated anthocyanins, have been investigated as functional additives in packaging systems, the use of its sepal mucilage as the primary film‐forming matrix has not been reported to date. HSSM offers distinct advantages for this purpose, including intrinsic bioactive compounds (phenolic acids, anthocyanins, and flavonoids) that may provide antioxidant activity without the need for additional extracts. However, as a hydrophilic polysaccharide, HSSM also has high water affinity and moisture sensitivity, which makes investigation of its combination with structural polysaccharides such as CMC particularly relevant for improving film performance. Specifically, the mechanical strength, film‐forming performance, structural and barrier properties, and functional properties of HSSM–CMC composite films, as well as the potential contribution of CMC to improving these properties, have not been sufficiently characterized. Determining the potential of HSSM–CMC composite films for sustainable active food packaging is therefore important. The present study aimed to investigate this research gap by developing and characterizing HSSM‐CMC composite films, including their physicochemical, mechanical, thermal, and functional (antioxidant and antimicrobial) properties, to evaluate their potential as a sustainable and promising biopolymer source for active food packaging applications.

## 2 Materials and Methods

### 2.1 Materials

The dried leaves of sour tea plant ( *Hibiscus sabdariffa* ) were gathered Research Center for Medicinal and Ornamental Plants of Sistan and Baluchistan University (Zahedan, Iran). Other material, including calcium nitrate.6H2O (utilized to equilibrate films at 50% RH), glycerol and CMC (utilized to make film‐forming dispersions) and were obtained from the Merck Corporation. 2, 2‐diphenyl‐1‐ picrylhydrazyl (DPPH) was bought from Sigma Chemical Co. All the other reagents used of an analytical grade.

### 2.2 Methods

#### 2.2.1 Extraction HSS ( *Hibiscus sabdariffa* Sepal) Mucilage

**FIGURE 1:**
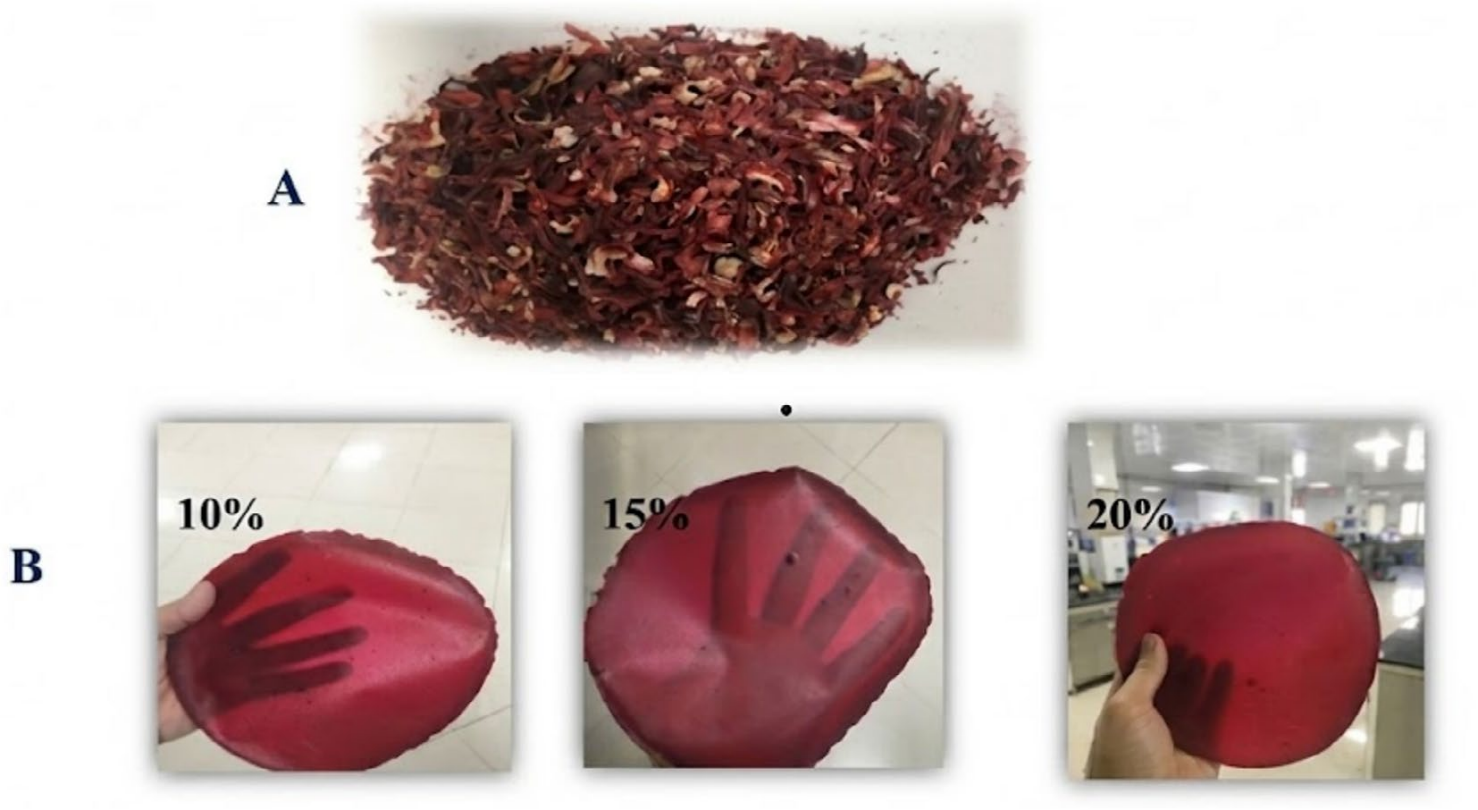
(A) *Hibiscus sabdariffa* sepal before swelling in water, (B) HSSM‐CMC films prepared with different glycerol contents (10%, 15%, and 20% w/w).

The mucilage was extracted using a multi‐step procedure. Prior to extraction, *Hibiscus sabdariffa* leaves were air‐dried in the shade under ambient laboratory conditions (25°C ± 2°C) and ground into powder using an electric grinder (Figure 1A). The powdered leaves were passed through a 60‐mesh sieve to obtain a uniform powder and remove foreign particles. The extraction followed a modified version of the method described by Beigomi et al. (2018). In brief, powdered leaves were mixed with distilled water at a 1:20 (w/v) ratio (25 g powdered leaves in 500 mL distilled water) and kept at 25°C for 4 h, followed by an additional 12‐h standing period. The mixture was then stirred at 700 rpm for 1 h using a magnetic stirrer and subsequently filtered. Finally, acetone was added to the filtrate at a ratio of 1:3 (filtrate: acetone, v/v) to precipitate and recover the mucilage, which was then dried for further use (Beigomi et al. 2018).

#### 2.2.2 Analytical Methods

The extraction yield was calculated as the ratio of the dry weight of mucilage to the initial weight of the seeds. Ash and moisture contents were determined following the AOAC protocols. Ash content was measured gravimetrically after incinerating the samples at 500°C for 12 h in a muffle furnace. Protein content was quantified using the Kjeldahl method according to AOAC (1990) guidelines, applying a conversion factor of 6.25 for total nitrogen (International A. 1990). Carbohydrate content was estimated by subtracting the measured amounts of moisture, protein, fat, and ash from the total dry matter.

#### 2.2.3 Preparation of HSSM Film

Films were prepared from solutions containing 1.25% (w/v) mucilage, which was identified as the optimal concentration among the tested range (0.5%, 0.75%, 1%, 1.25%, and 1.5%). Solutions with less than 1.25% mucilage produced thin and fragile films, whereas higher concentrations resulted in a gel‐like consistency. The method described by Beigomi et al. (2018) was employed to form the films (Beigomi et al. 2018). Glycerol was added as a plasticizer at 10%, 15%, and 20% (w/w) under continuous stirring at 750 rpm, and the mixture was homogenized at 12,000 rpm for 5 min to achieve a uniform polymeric dispersion. The solution was then degassed under vacuum oven Sheldon Manufacturing Inc., Cornelius, OR, USA at 37°C ± 2°C for 7 min to remove air bubbles. CMC was incorporated as a structural polymer to improve film‐forming ability and provide additional intermolecular interactions within the HSSM‐CMC matrix. The selected CMC concentration (1%, w/w) was consistent with previous studies on CMC‐based edible films (Ballesteros et al. 2018; Ghanbarzadeh and Almasi 2011). In the present study, CMC was maintained at a low effective concentration to provide structural support while maintaining the functional and bioactive contribution of HSSM within the film matrix. A 1% (w/w) CMC solution was prepared based on the procedure of (Ghanbarzadeh and Almasi 2011). Subsequently, the mucilage–glycerol mixture was blended into the CMC solution to form a homogenous network. Films were cast on Teflon plates and allowed to dry at room temperature (21°C ± 2°C) under 35% relative humidity for 48 h (Figure 1B). After drying, the films were peeled off and conditioned for 48 h in a desiccator containing a saturated solution of Ca(NO3)_2_·6H_2_O (50% ± 2% RH) prior to further characterization.

### 2.3 Film Characterization

#### 2.3.1 Film Thickness and Density

Film thickness was measured using a digital micrometer with a precision of 0.001 mm. The density of the films (ρ_*s*_) was calculated based on the dry weight and measured geometrical dimensions using the following equation:

(1) $$\rho_{s}=M/A\times \delta$$

where A represents the film area (4 cm × 4 cm), M is the dry mass of the film (g), *δ* is the measured thickness (mm), and ρ_*s*_ denotes the density of the dry matter in the film (g/cm^3^) (Ghanbarzadeh and Almasi 2011).

#### 2.3.2 Moisture Content

The samples were cut into (3 × 3 cm.) specimens and weighed to assess the moisture content of films. The samples were then dried in an oven at 103°C until constant weight and weighed again (dry weight). The films' moisture content (MC) was calculated using the following equation ((2)):

(2) $$\mathrm{MC}\;\left(\%\right)=\left(\frac{M_{i}-M_{f}}{M_{i}}\right)\times 100$$

where M_*i*_ and M_*f*_ are the masses of the initial and the dried samples respectively (Chiumarelli and Hubinger 2014).

#### 2.3.3 Film Solubility in Water

Water solubility (WS) of the films was determined to assess the stability of the polymeric network in aqueous conditions. Film samples (3 × 3 cm) were first dried at 103°C ± 2°C for 24 h until constant weight (W0) and weighed. The dried films were then submerged in 80 mL of distilled water and stirred at 200 rpm for 1 h at room temperature. After immersion, the insoluble film fraction was dried again at 103°C ± 2°C to constant weight (W1), and the final weight was recorded. Water solubility was calculated using the following equation:

(3) $$\mathrm{WS}\;\left(\%\right)=\left(\frac{W_{i}-W_{f}}{W_{i}}\right)\times 100$$

where *W*_i_ is the initial weight of film and *W*_*f*_ is the weight of the dried undissolved film (Gontard and Guilbert 1994).

#### 2.3.4 Free Radical Scavenging Assay

The antioxidant capacity of the biodegradable polymer films was assessed using the DPPH (2, 2‐diphenyl‐1‐picrylhydrazyl) radical‐scavenging assay, following the procedure of Brand‐Williams et al. (1995). Briefly, 25 mg of film sample was dissolved in 5 mL of distilled water. Then, 0.1 mL of this solution was combined with 3.9 mL of a 0.1 mM DPPH solution prepared in methanol and incubated in the dark for 30 min. The absorbance of the mixture was recorded at 517 nm using a Perkin‐Elmer spectrophotometer (Brand‐Williams et al. 1995). The radical scavenging activity (%) was calculated as follows:

(4) $$\text{radical scavenging activity}\;\left(\%\right)=\frac{A_{\text{reference}}-A_{\text{sample}}}{A_{\text{reference}}}\times 100$$

where *A*_sample_ represents the absorbance of the sample solution and *A*_reference_ represents the absorbance of DPPH solution without the addition of the film.

#### 2.3.5 Water Vapor Permeability (WVP)

The water vapor permeability of the biodegradable polymer films was measured based on the ASTM E96‐00 standard (International A 2000). Circular aluminum cups containing 3.0 g of anhydrous calcium chloride (providing 0% relative humidity) were covered and sealed with the prepared film samples. The cups were then weighed and placed inside a desiccator containing a saturated sodium chloride solution to maintain a controlled humidity gradient. The driving force (1753.55 Pa) was defined as the difference in partial vapor pressure across both sides of the film. The weight gain of each cup was recorded every six‐hour intervals over a 24‐h period to determine the moisture transmission rate. WVP was calculated according to the following equation:

(5) $$\mathrm{WVP}=\frac{{∆}_{m}}{{∆}_{\mathrm{tA}}}.\frac{x}{{∆}_{p}}$$

where ∆_m_/∆_t_ denotes the rate of water vapor gain (g/s), A represents the exposed film area (m^2^), *X* is the film thickness (mm), and ∆_*p*_ indicates the difference in partial vapor pressure (Pa).

#### 2.3.6 Thermal Analysis

The thermal characteristics of the developed biodegradable polymer films were assessed using differential scanning calorimetry (DSC). Approximately 7 mg of each sample was heated from −100°C to 200°C at a constant rate of 10°C per minute. An empty aluminum pan served as the reference throughout the analysis (Khazaei et al. 2014).

#### 2.3.7 Mechanical Properties

The mechanical properties of the biodegradable polymer films were evaluated by measuring tensile strength and elongation at break (EB) using a texture analyzer M350‐10CT (Testometric Co. Ltd., Rochdale, Lancashire, England) in accordance with ASTM D882‐02 (2002). Film strips of uniform dimensions were tested under controlled environmental conditions (25°C ± 2°C, 50% ± 2% RH). Each test was conducted in triplicate to ensure accuracy and reproducibility of the results (International A 2002).

#### 2.3.8 Color Measurement

The color properties of the biodegradable polymer films were analyzed using a CIE colorimeter. Prior to measurement, the device was calibrated using a standard white plate (L* = 93.49, a* = 0.25, b* = 0.09). The samples were then placed on the plate, and the lightness (*L*)*, red–green (*a*)* and yellow–blue (b*) parameters were recorded. The total color difference (ΔE) was calculated according to equation (6)

(6) $$∆E=\sqrt{{\left(L*-L\right)}^{2}+{\left(a*-a\right)}^{2}+{\left(b*-b\right)}^{2}}$$

(7) $$\mathrm{WI}=100-\sqrt{{\left(100-L\right)}^{2}+{\left(a\right)}^{2}+{\left(b\right)}^{2}}$$

where L*, a* and b* are the color parameter values of the standard white plate (L* = 93.49, a* = −0.25 and b* = −0.09) and *L*, *a* and *b* are the color parameter values of the sample (Sadeghi‐Varkani et al. 2018).

#### 2.3.9 Antibacterial Activity of the Film

The antimicrobial potential of the optimized film was evaluated against six representative bacterial strains using the agar disc diffusion method (Seydim and Sarikus 2006). The bacterial strains tested included *Salmonella typhimurium* (ATCC 14028), *Staphylococcus aureus* (ATCC 25923), *Escherichia coli* (ATCC 25922), *Escherichia coli* O157:H7 (NCTC 12900), *Bacillus cereus* (ATCC 10876), and *Listeria monocytogenes* (ATCC 7644). Each strain was cultured in brain heart infusion (BHI) broth and incubated at 37°C until reaching the exponential growth phase. The turbidity of the bacterial suspension was adjusted to match a 0.5 McFarland standard, approximately 1.5 × 10^8^ CFU/mL.

For the agar diffusion assay, 0.1 mL of each bacterial culture (10^8^ CFU/mL) was spread onto BHI agar plates. The polymer films were cut into discs of 8 mm diameter and placed on the inoculated agar surface. After incubation at 37°C for 24 h, the inhibition zones surrounding the film discs were measured with a caliper to the nearest 0.02 mm. The clear zone area was calculated by subtracting the disc area from the total inhibition zone. Standard antibiotics, including gentamicin (20 μg/disc), tetracycline (30 μg/disc), vancomycin (30 μg/disc), and erythromycin (15 μg/disc), were used as positive controls.

#### 2.3.10 Surface Morphology

The surface and cross‐sectional morphologies of the dried biodegradable polymer films were investigated using scanning electron microscopy (SEM) (KYKY EM‐3200, China). Film samples were mounted on metal stubs, sputter‐coated with a thin gold layer, and observed under vacuum conditions at an accelerating voltage of 20 kV (Khazaei et al. 2014).

## 3 Statistical Analysis

Each test was performed in triplicate. Prior to statistical analysis, the normality of data distribution was assessed using the Kolmogorov–Smirnov test, and the homogeneity of variances was evaluated using Levene's test. The obtained data were analyzed using SPSS software version 20. Analysis of variance (ANOVA) and Duncan's multiple range test (*p* < 0.05) were applied to identify significant differences among samples. All charts and tables were prepared using Sigma Plot version 12.

## 4 Results and Discussion

### 4.1 Proximate Compositions of *Hibiscus sabdariffa* Sepal Mucilage

Analysis of the chemical composition of HSSM revealed that it consisted of 3.4% protein, 7.2% moisture, 13.2% ash, 0.3% fat, and 75.90% carbohydrates. The overall extraction yield was determined to be 9.06%.

### 4.2 Physical Properties of the Films

The effect of different glycerol contents on the thickness of HSSM‐based biodegradable polymer films is presented in Table 1. Film thickness varied between 0.019 and 0.029 mm and increased with higher glycerol content. This can be attributed to the higher moisture absorption and plasticizing capacity of glycerol, causing swelling and expansion of the polymer matrix. Glycerol molecules penetrate between macromolecular chains, enlarging the network and increasing film thickness. Similar results were reported by Dick et al. (2015) for chia seed mucilage films and Sadeghi et al. (2018) for balangu seed mucilage films (Dick et al. 2015; Sadeghi‐Varkani et al. 2018).

**TABLE 1:** Physical properties of *Hibiscus sabdariffa* sepal mucilage (HSSM) biodegradable polymer film incorporated with various glycerol concentrations as plasticizer.

Glycerol content (w/w)	Density (g/cm^3^)	Solubility (%)	Moisture (%)	Thickness (mm)	Water vapor permeability (× 10^−10^gs^−1^ m^−1^ pa^−1^)
Without plasticizer	1.61 ± 0.01^a^	24.42 ± 0.09^d^	11.42 ± 0.090^d^	0.0195 ± 0.005^c^	1.21 ± 0.015^c^
10	1.45 ± 0.2^b^	35.33 ± 0.59^c^	18.33 ± 1.15^c^	0.0235 ± 0.005^b^	1.35 ± 0.025^c^
15	1.41 ± 0.01^c^	40.22 ± 0.25^b^	22.52 ± 0.53^b^	0.024 ± 0.012^b^	1.56 ± 0.051^b^
20	1.2 ± 0.02^d^	50.31 ± 0.11^a^	30.01 ± 1.15^a^	0.029 ± 0.0010^a^	2.32 ± 0.14^a^

*Note:* Means within each column with same letters are not significantly different (*p* < 0.05). Data are means ± SD.

On the other hand, the density of all film samples decreased with increasing glycerol content. The density of HSSM films (Table 1) ranged between 1.2 and 1.61 g/cm^3^. Similar findings were reported in our previous work for film‐based *Dracocephalum moldavica* seed mucilage, where the density decreased from 0.92 to 0.89 g/cm^3^with increasing glycerol content from 10% to 40% (w/w) (Beigomi et al. 2018). Moreover, Seyedi et al. (2014) indicated that the density of *Lepidium perfoliatum* seed gum‐based films decreased from 0.93 to 0.88 g/cm^3^, with increasing glycerol content, however, no significant decrease was observed for higher GLY concentration (*p* > 0.05) (Seyedi et al. 2014).

This decrease in density with increasing glycerol content can be attributed to the plasticization effect of glycerol, which inserts between polymer chains and increases the free volume of the network, reducing matrix compactness without a proportional increase in mass.

As shown in Table 1, moisture content of the HSSM‐based biodegradable polymer films increased from 11.42% to 30.01% with increasing glycerol, in agreement with (Osés et al. 2009). The hydrophilic nature and hydroxyl groups of glycerol enable strong water binding, acting as a moisture‐retaining agent within the polymer matrix. Similar trends were reported for kefiran‐ and psyllium‐based polymer films with higher glycerol contents (Ghasemlou et al. 2011; Ahmadi et al. 2012).

Water solubility of the films (Table 1) also increased with glycerol content, indicating weaker biopolymer interactions and greater hydrophilicity. The expanded free volume between chains enhances water diffusion within the polymer matrix, thus increasing solubility. Comparable behavior was observed for chia seed mucilage films, where solubility increased significantly (*p* < 0.05) from 52.74% to 84.50% (Dick et al. 2015).

### 4.3 Water Vapor Permeability (WVP)

The results in Table 1 show that increasing glycerol content raised the water vapor permeability (WVP) from 1.21 to 2.32 gs^−1^ m^−1^ Pa^−1^ × 10^−10^. The number of OH groups in a molecule directly affects its WVP. Higher glycerol levels weaken intermolecular interactions between polymer chains, increasing free volume and segmental mobility, which facilitates water molecule diffusion and thus increases WVP. At high concentrations, glycerol clusters may also disrupt the film matrix, further enhancing permeability.

Water solubility also influences WVP since hydrophilic films absorb water vapor, leading to condensation and partial dissolution, allowing water molecules to diffuse through. Low‐molecular‐weight glycerol penetrates polymer chains, enlarging micro‐pores and further increasing vapor permeability (Seyedi et al. 2014).

Similarly, Yang and Paulson (2000) reported that WVP of gellan‐based films increased with higher glycerol levels due to greater chain spacing and enhanced vapor diffusion (Yang and Paulson 2000). Sadeghi‐Varkani et al. (2018) also observed a significant rise in WVP (4.36%–18.38%) in Balangu seed mucilage films as glycerol content increased from 10% to 60% (*p* < 0.05) (Sadeghi‐Varkani et al. 2018).

Surface wettability is another important factor affecting the interaction between biopolymer packaging materials and water molecules. The wettability behavior of polysaccharide‐based films is influenced by the chemical nature of the constituent polymers and plasticizers, particularly the presence of hydrophilic functional groups (Eranda et al. 2025). In the present HSSM‐CMC system, both glycerol (10%–20%, w/w) and CMC (fixed at 1%, w/w) contribute hydroxyl/carboxyl groups to the film matrix, which may affect interactions between the film structure and water molecules. Consistent with this behavior, increasing glycerol content was accompanied by higher moisture content, water solubility, and WVP values, indicating an increased affinity of the film matrix toward water. These findings highlight the role of hydrophilic components in influencing the water‐related behavior of HSSM‐CMC films.

The increase in WVP and WS with increasing glycerol content is consistent with the hydrogen bonding and increased free volume mechanism described in Section 4.6. Hydrophilic plasticizers such as glycerol introduce additional hydroxyl groups into the film matrix, which can form hydrogen bonds with water molecules and increase the hydrophilicity of the polymer network, thereby promoting water vapor diffusion (Eslami et al. 2023; Nur Hanani et al. 2013; Cerqueira et al. 2012). From a practical perspective, higher‐glycerol films may be less suitable for high‐moisture or high‐water‐activity food packaging and may instead be better suited for lower‐moisture, dry food products. Future strategies, including the incorporation of hydrophobic compounds, crosslinking, or blending with more hydrophobic polymers, could help achieve a better balance between barrier properties and flexibility while preserving the effect of glycerol in enhancing film flexibility.

### 4.4 Antioxidant Activity of the HSSM Film—DPPH Method

The results indicated that the DPPH radical scavenging activity of biodegradable polymer film prepared from HSSM without any plasticizer was 8.22%. Antioxidants are compounds that help protect polymer systems by minimizing oxidative reactions. Although synthetic antioxidants are widely used in food‐related applications due to their high efficiency and low cost, their use in polymeric edible films is limited because of potential toxic and hazardous effects. The DPPH assay is a widely accepted and reliable method for evaluating antioxidant activity in polymeric systems. This technique is based on the color change of the DPPH solution from purple to yellow, which occurs when antioxidants donate hydrogen atoms and reduce the DPPH free radicals (Brand‐Williams et al. 1995).

The observed antioxidant activity is consistent with the phenolic acid and anthocyanin content of *Hibiscus sabdariffa* , whose phenolic hydroxyl groups can donate hydrogen atoms to neutralize DPPH free radicals. Similar antioxidant behavior has also been reported for other plant mucilage‐based films (Beigomi et al. 2018; Cerqueira et al. 2012), suggesting the potential of HSSM as a natural antioxidant source for active food packaging applications. As this activity was assessed using a single assay (DPPH), further confirmation with a complementary method (e.g., ABTS or FRAP) is recommended in future studies.

### 4.5 Thermal Properties

The thermal behavior of the biodegradable polymer films was examined using differential DSC to gain insights into the interactions between the polymers and plasticizers. During heating, polymeric materials experience various phase transitions, each associated with distinct thermal characteristics. The glass transition temperature (Tg) indicates the temperature at which the material transitions from a rigid, glassy state to a more flexible, rubbery state. Below Tg, the films exhibit hardness and brittleness, whereas above Tg, they display increased softness and flexibility. Tg is also an indicator of polymer‐plasticizer compatibility. Another important transition is melting, representing the change from crystalline to amorphous structure (Sadeghi‐Varkani et al. 2018; Yang and Paulson 2000).

The results of thermal properties are shown in Table 2. Since only one glass transition temperature (Tg) was observed for all films, glycerol can be considered a compatible plasticizer. According to Ghasemlou et al. (2011), when two Tg values appear, the plasticizer and polymer are immiscible, representing two separate phases (Ghasemlou et al. 2011). Increasing glycerol content reduced Tg values due to the plasticizing effect of glycerol molecules. Based on the free volume theory, glycerol molecules penetrate polymer chains, expand the free volume, and increase chain mobility, which lowers Tg.

**TABLE 2:** Tensile strength (TS), elongation at break (EB) and thermal properties for *Hibiscus Sabdariffa* sepal mucilage (HSSM) biodegradable polymer film incorporated with various glycerol concentrations as plasticizer.

Glycerol content (w/w)	TS (MPa)	EB (%)	Tm (°C)	Tg (°C)
Without plasticizer	—	—	129.6 ± 0.65^a^	—
10	20.40 ± 1.55^a^	17.67 ± 0.32^c^	127.02 ± 0.19^b^	56.84 ± 1.39^a^
15	16.54 ± 0.70^b^	25.28 ± 1.20^b^	113.8 ± 0.65^c^	46.40 ± 1.90^b^
20	10.51 ± 0.62^c^	27.91 ± 0.49^a^	112.2 ± 0.75^d^	42.24 ± 1.11^c^

*Note:* Means within each column with same letters are not significantly different (*p* < 0.05). Data are means ± SD.

The Tg values of HSSM‐CMC films (42.24°C–56.84°C) were higher than typical ambient and refrigeration temperatures, indicating that the films remained in a glassy state during common food storage conditions. This behavior agrees with the higher tensile strength and lower EB observed at lower glycerol concentrations, as the reduced plasticizer content limits polymer chain mobility. With increasing glycerol content, the decrease in Tg indicates increased polymer chain mobility, resulting in greater film flexibility and higher elongation at break. Therefore, adjusting the plasticizer level may help control the flexibility of HSSM‐CMC films for specific packaging applications.

Khazaei et al. (2014) also reported one Tg in basil seed gum films, and the melting point decreased due to a reduction in hydroxyl groups that form hydrogen bonds among polymer chains (Khazaei et al. 2014). Similarly, Seyedi et al. (2014) found that glycerol in *Lepidium perfoliatum* seed gum films reduced Tg by decreasing polysaccharide interactions and stabilizing the polymer network (Seyedi et al. 2014). Higher glycerol contents required less enthalpy to break interchain bonds. An increase in glycerol from 40% to 60% (w/w) decreased Tg as a result of greater free volume and chain mobility. Tg values were inversely related to moisture content, which acts as a softener and enhances molecular mobility, leading to lower Tg values (Dick et al. 2015).

### 4.6 Mechanical Properties

The findings of the mechanical characteristics of films made with different glycerol contents are presented in Table 2. As shown, the EB increased, whereas tensile strength (TS) decreased with increasing glycerol content. The TS decreased from 20.40 to 10.51 MPa, while EB increased from 17.67% to 27.91%, indicating that increasing glycerol content enhances film extensibility while reducing resistance.

**FIGURE 2:**
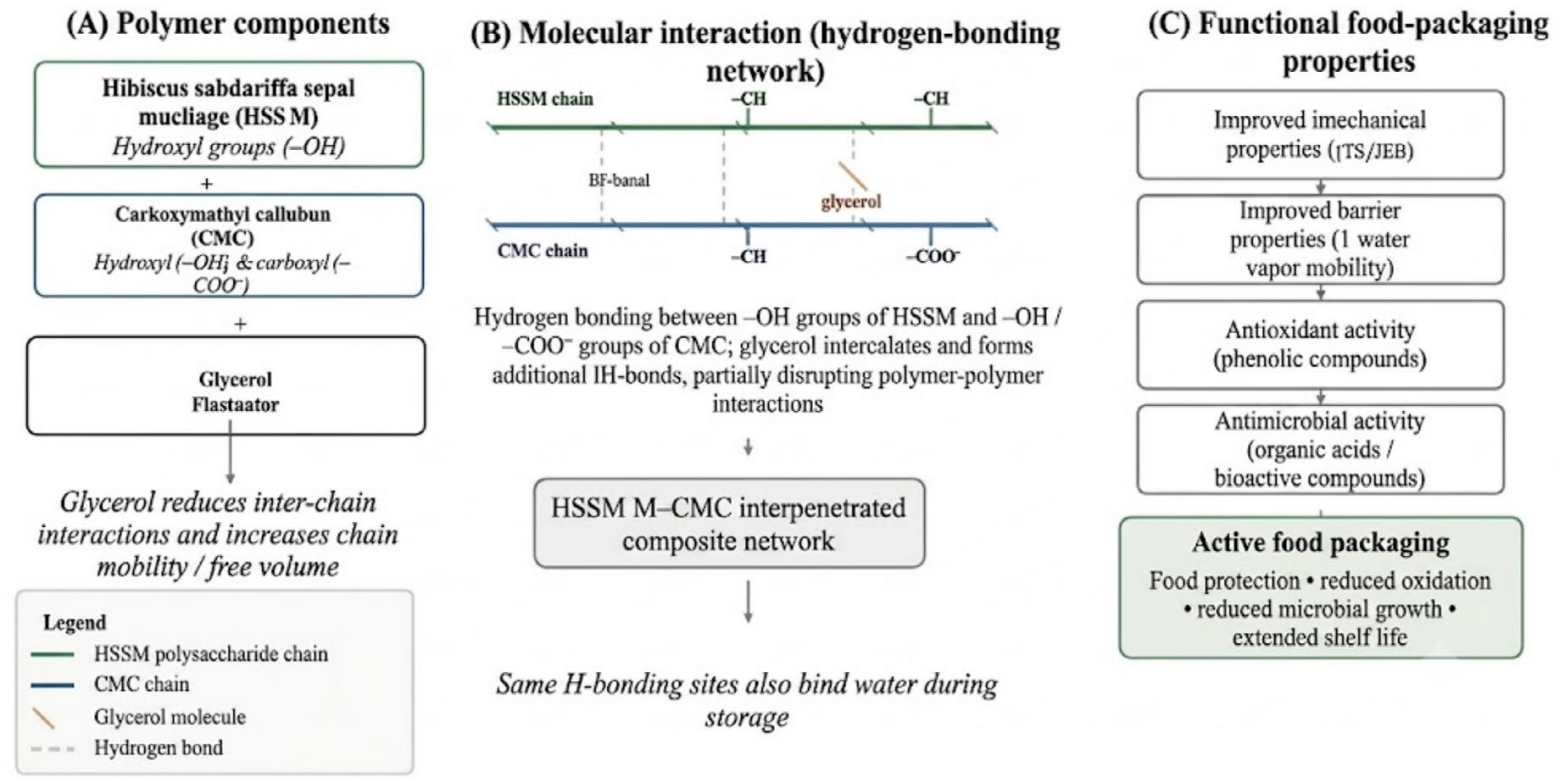
Proposed mechanism of interaction among HSSM, CMC, and glycerol in the composite film, and its relation to functional food‐packaging properties.

The proposed interaction mechanism among the composite phases involves hydrogen‐bond formation between the hydroxyl groups of HSSM polysaccharide chains and the hydroxyl/carboxyl groups of CMC chains, forming a hydrogen‐bonded polymer network. Glycerol molecules are incorporated within this network, forming additional hydrogen bonds with both polysaccharide chains that partially disrupt polymer‐polymer interactions, thereby increasing free volume and chain mobility (Thakhiew et al. 2010). This interaction mechanism helps explain the observed TS/EB trends and is expected to influence the film's performance as a barrier material for food packaging, since the same hydrogen‐bonding sites involved in polymer‐polymer and polymer‐glycerol interactions are also available to interact with water molecules during food storage (Figure 2). This behavior is consistent with the moisture sensitivity discussed in Section 4.3.

Jouki, Mortazavi, et al. (2013), reported that in cress seed carbohydrate gum‐based films, glycerol caused a significant difference in TS and EB. In films containing 50% (w/w) glycerol, TS decreased by 42%, while EB increased markedly to 115% (*p* < 0.05). Thus, higher glycerol content enhanced polymer chain mobility, improving film extensibility while reducing mechanical resistance (Jouki, Mortazavi, et al. 2013).

Thakhiew et al. (2010) also reported that in chitosan‐based films, glycerol content significantly affected TS and EB. Films without glycerol exhibited the highest TS and lowest EB, whereas the addition of glycerol resulted in a more flexible polymer network. This effect is attributed to glycerol intercalation within the polymer matrix, which disrupts intermolecular interactions and increases chain mobility, thereby enhancing flexibility (Thakhiew et al. 2010).

Similar glycerol‐dependent trends have been reported for other seed mucilage‐ and polysaccharide‐based composite films. In basil seed gum films, TS decreased from 31.69 to 18.14 MPa while EB increased from 18.55% to 39.37% as glycerol content rose from 0% to 50% (w/w) (Khazaei et al. 2014). A comparable pattern was observed for chia seed mucilage films, in which TS decreased from 17.75 to 9.44 MPa and EB increased from 1.93% to 15.89% across a glycerol range of 25%–75% (w/w) (Dick et al. 2015). For Balangu seed mucilage films, the optimal formulation (1.2% mucilage, 35% glycerol) yielded a TS of 15.37 MPa and a markedly higher EB of 90.27% (Sadeghi‐Varkani et al. 2018). The TS values obtained for the present HSSM‐CMC films (10.51–20.40 MPa) are comparable to those reported for seed mucilage‐based films, exceeding those of chia and Balangu mucilage films while remaining below the upper range of basil seed gum films. This is consistent with the strengthening effect of CMC incorporation, which has similarly been shown to increase the tensile strength of starch‐based films without a significant loss of flexibility (Ghanbarzadeh et al. 2011). Relative to commercial CMC‐based films, Haqiqi et al. (2021) reported TS values of 30.27–33.87 MPa for pure CMC and CMC‐lignin composite films, which exceed the TS values obtained in the present study, while their EB values (8.33%–24.47%) were comparable to those of the present HSSM‐CMC films. Compared with conventional petroleum‐based packaging plastics, the TS of the present films falls within the range reported for low‐density polyethylene (8–10 MPa) and the lower range of high‐density polyethylene (19–31 MPa) (Haqiqi et al. 2021), supporting the potential of the HSSM‐CMC system as a biodegradable alternative for flexible, non‐structural food packaging applications.

The free volume theory of plasticization, introduced in Section 4.2 to explain the glycerol‐dependent decrease in film density, provides a consistent mechanism for the observed changes in several film properties. The increased free volume facilitates water molecule diffusion through the polymer network, contributing to the increased WVP. In addition, the reduced intermolecular interactions and enhanced chain mobility associated with increased free volume result in decreased TS and increased EB, as well as a reduction in Tg due to greater molecular mobility.

### 4.7 Color Measurement

Among the key factors influencing the optical properties of the polymeric films are the CIE color parameters (L*, a*, b*), total color difference (∆E), and whiteness index (WI), summarized in Table 3. As seen, increasing glycerol content increased lightness (L*), blue–yellow (b*), green–red (a*), and WI values, while ∆E decreased. Due to the inherent color of *Hibiscus sabdariffa* sepal mucilage, the films exhibited a reddish hue, with the a* index increasing at higher glycerol contents. The observed increase in L and WI values with glycerol content may be related to the improved matrix uniformity observed by SEM (Section 4.9), which can reduce light scattering from structural heterogeneities.

**TABLE 3:** Color values including, L, a, and b, total color difference (∆E) and whiteness index (WI) *Hibiscus sabdariffa* sepal mucilage (HSSM) biodegradable polymer film as a function of glycerol concentration.

Glycerol content (w/w)	∆*E*	b	a	L	WI
Without plasticizer	14.72 ± 0.27^a^	4.20 ± 0.1^d^	1.89 ± 0.29^b^	79.44 ± 0.29^d^	78.95 ± 0.24^c^
10	13.96 ± 0.27^b^	4.60 ± 0.1^c^	2.60 ± 0.27^a^	80.47 ± 0.27^c^	79.73 ± 0.22^b^
15	13.15 ± 0.36^c^	5.26 ± 0.15^b^	2.55 ± 0.054^ab^	81.62 ± 0.38^b^	80.86 ± 0.35^a^
20	12.60 ± 0.54^c^	5.76 ± 0.15^a^	3.04 ± 0.019^a^	82.52 ± 0.60^a^	81.17 ± 0.69^a^

*Note:* Means within each column with same letters are not significantly different (*p* < 0.05). Data are means ± SD.

Khazaei et al. (2014) and Ahmadi et al. (2012) reported that in psyllium seed‐based films, increasing glycerol content led to higher brightness (L*) and blue–yellow (b*) values, while green–red (a*) values decreased. Additionally, the whiteness index (WI) increased significantly (*p* < 0.05) and ∆E decreased, resulting in films that were slightly greenish‐yellow but remained transparent (Khazaei et al. 2014; Ahmadi et al. 2012). The slight increase in a* and b* values with glycerol content is more difficult to attribute to a single cause. Considering the organic acid content of *Hibiscus sabdariffa* sepals, anthocyanins may remain mainly in the red flavylium cation form across the studied glycerol concentrations and this equilibrium is mainly determined by pH rather than moisture content alone. It is therefore more likely that the observed color change reflects glycerol's effects on pigment solubility and mobility within the film matrix, its influence on anthocyanin–polymer interactions, or changes in the local polarity of the pigment microenvironment, rather than a direct change in the flavylium–carbinol equilibrium. Further work would be needed to clarify the specific mechanism underlying this observation.

### 4.8 Antimicrobial Properties of HSSM Film

The antimicrobial activity of HSSM‐based polymeric films was evaluated using the agar disk diffusion method against *E. coli* , *S. aureus* , *L. monocytogenes* , *E. coli O157:H7*, *S. typhimurium* , and *B. cereus* . Gentamicin, tetracycline, vancomycin, and erythromycin served as positive controls. The inhibition zone diameters produced by the film disks are summarized in Table 4.

**TABLE 4:** Inhibition zone diameters yielded by *Hibiscus sabdariffa* sepal mucilage (HSSM) biodegradable polymer film disks.

Inhibition zone diameters (mm)						
	*S. aureus*	*L. monocytogenes*	*E. coli*	*E. coli O157:H7*	*S. typhimurium*	*Bacillus cereus*
HSSM film	13.66 ± 0.15^e^	10.16 ± 0.25^e^	11.73 ± 0.0^c^	8.73 ± 0.55^d^	0^e^	15.63 ± 0.3^d^
Gentamicin	14.2 ± 0.18^d^	13.7 ± 0.21^d^	20.6 ± 0.13^b^	13.4 ± 0.23^c^	14.6 ± 0.16^b^	15.9 ± 0.31^c^
Tetracycline	25 ± 0.21^a^	23.49 ± 0.14^a^	22.3 ± 0.21^a^	29.25 ± 0.10^a^	9.2 ± 0.21^c^	10 ± 0.1^e^
Vancomycin	18 ± 0.10^c^	19 ± 0.13^c^	2 ± 0.21^d^	17 ± 0.19^b^	19.20 ± 0.26^a^	20.21 ± 0.21^a^
Erythromycin	24 ± 0.16^b^	23 ± 0.21^b^	0^e^	0^e^	4 ± 0.19^d^	17 ± 0.16^b^

*Note:* Reported values correspond to the mean ± standard deviation. Different letters in each column indicate a significant difference (*p* < 0.05).

The HSSM‐based polymeric films exhibited inhibitory effects against all tested microorganisms except *S. typhimurium* . Among the strains, *S. aureus and B. cereus* were more susceptible, while *S. typhimurium* showed lower sensitivity. These results are consistent with findings by Borrás‐Linares (2015), indicating that *Hibiscus sabdariffa* extracts are generally more active against Gram‐positive bacteria ( *S. aureus* and *B. cereus* ) than Gram‐negative bacteria ( *E. coli* and *S. enteridis*) (Borrás‐Linares 2015).

In general, Gram‐positive bacteria are more sensitive to plant‐derived compounds than Gram‐negative bacteria. It seems that the differences in their cell wall structures are the important reason for their sensitivity. The peptidoglycan outer membrane of Gram‐positive bacteria, together with other molecules such as teichoic acid and protein, allows hydrophobic molecules such as flavonoids and terpenoids to easily penetrate the cells and act on both the cell wall and within the cytoplasm (Cowan 1999).

HSSM‐based polymeric films, exhibiting confirmed antimicrobial activity, have the potential to serve as active polymer matrices by incorporating bioactive compounds. These films inhibit microbial growth through the controlled release of active molecules within the polymer network.

Although the inhibitory activity of HSSM films is lower than that of conventional antibiotics, the polymeric matrix offers advantages in sustained release and potential integration with other functional polymers for antimicrobial packaging applications.

Although the inhibitory activity of HSSM films is lower than that of conventional antibiotics, the polymeric matrix offers advantages in sustained release and potential integration with other functional polymers for antimicrobial packaging applications. Unlike antibiotics, which act on specific molecular targets at concentrations optimized for medical use, HSSM‐CMC films are intended for food‐contact packaging applications, where the controlled release of bioactive compounds and broad antimicrobial effects are more relevant.

It should be noted that the standard antibiotics included in this assay served as positive controls to confirm strain susceptibility and provide a reference for antimicrobial activity assessment, rather than as a direct reference for the film's antimicrobial activity. The antimicrobial activity of the HSSM‐CMC film, attributed to its phenolic compounds, including anthocyanins/flavonoids, likely results from broader antimicrobial mechanisms such as membrane disruption. Direct comparison of inhibition zone diameters between these clearly different classes of antimicrobial agents should therefore be evaluated with caution.

The development of composite biopolymer films incorporating bioactive natural compounds reflects a broader trend toward active and smart food packaging systems. Polysaccharide‐based platforms such as CMC have been increasingly combined with plant‐derived bioactive extracts to enhance antimicrobial and antioxidant functionality; for example, CMC composite films enriched with pomegranate peel extract have recently demonstrated enhanced antibacterial and antioxidant activity alongside improved barrier and mechanical properties (Baniasadi et al. 2025). Similarly, gelatin–chitosan films enriched with plant extracts have been explored for extending the shelf life of perishable seafood products through combined antimicrobial and antioxidant activity (Eranda et al. 2024). The antibacterial activity observed in the present HSSM‐CMC films is consistent with this broader trend toward plant‐polysaccharide‐based active packaging systems.

### 4.9 Film Microstructure

**FIGURE 3:**
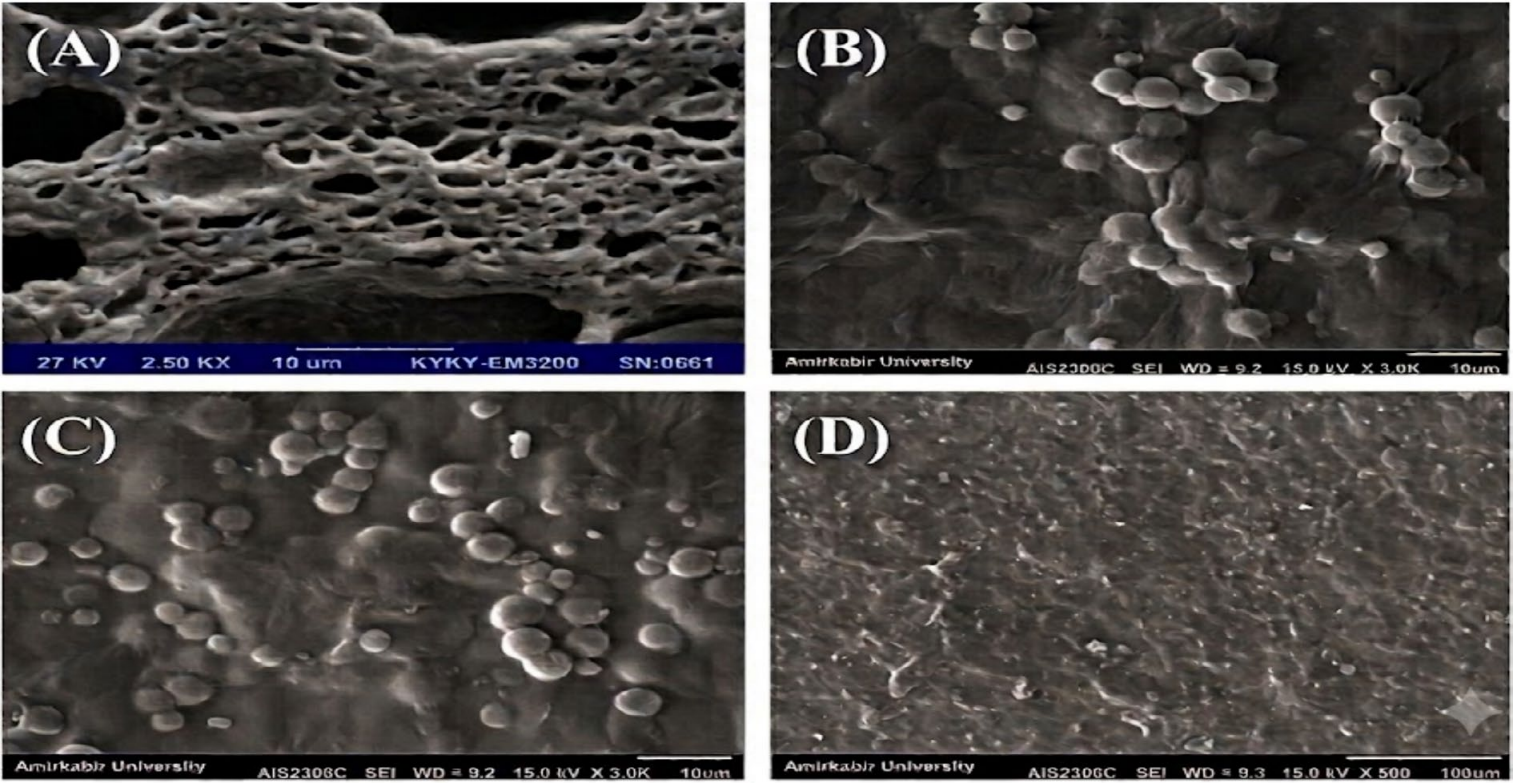
Scanning electron microscopic images of HSSM‐based biodegradable polymer films (A) unplasticized, (B–D) plasticized with 10%, 15%, 20% glycerol respectively.

Figure 3 illustrates the surface (left) and cross‐sectional (right) micrographs of the HSSM‐based polymeric films. The images indicate that films containing glycerol as a plasticizer exhibited smooth and uniform cross‐sections without cracks or fissures, in contrast to films prepared without glycerol, indicating that the cross‐section itself has a cohesive structure and smooth uniform surface morphology. No significant structural differences were observed among films with varying glycerol contents. The absence of holes or fractures on the surface of glycerol‐containing films indicates acceptable miscibility and compatibility of glycerol within the polymer matrix.

Similar observations have been reported by Jouki, Mortazavi, et al. (2013), who found that glycerol‐plasticized films displayed smooth, homogeneous surfaces without significant cracks, fractures, or openings, and that differences between various glycerol contents were minimal (Jouki, Mortazavi, et al. 2013). Dick et al. (2015) also reported comparable findings for chia seed mucilage‐based films incorporating carboxymethyl cellulose, highlighting the role of the polymer matrix in maintaining structural integrity (Dick et al. 2015).

Although SEM micrographs did not reveal visually resolvable differences among films with varying glycerol content, the mechanical and barrier properties of these films varied systematically with glycerol content. This difference can be explained by the distinct scales evaluated by these techniques: SEM resolves microstructural surface and cross‐sectional morphology, whereas glycerol's plasticizing effect acts primarily at the molecular scale through hydrogen‐bonding interactions and free‐volume changes discussed in Section 4.6. Therefore, the absence of visible microstructural differences is not inconsistent with the proposed molecular mechanism and is consistent with the expected plasticizing effect of glycerol.

## 5 Study Limitations and Future Research Directions

Although the optimized HSSM‐CMC film was comprehensively characterized in terms of its physicochemical, mechanical, thermal, antioxidant, and antimicrobial properties, some properties associated with surface characteristics, environmental stability, and practical application still need to be investigated. Among these aspects, the biodegradation behavior of HSSM‐CMC films remains to be investigated. Experimental biodegradation studies, such as soil incubation or composting tests with periodic weight‐loss and structural monitoring, would provide direct evidence to support the film's biodegradable nature and represent an important direction for future work. Water contact angle (WCA) measurement combined with AFM‐based surface roughness analysis, which were not conducted in the present study, are also recommended to provide a more comprehensive evaluation of surface wettability and its relationship with surface microstructure. Moreover, investigating the stability of the films under different storage conditions, including variations in pH, humidity, and light exposure, would be valuable for predicting their behavior during food packaging applications. Further studies could also evaluate the ability of the films to maintain their structural and functional properties after exposure to different environmental conditions. In addition, assessing antioxidant activity across all glycerol formulations (e.g., with a complementary assay such as ABTS or FRAP) and examining the relationship between moisture content and mechanical properties would help clarify the influence of formulation composition on film characteristics. Furthermore, substituting conventional organic solvents (e.g., acetone, used in the mucilage precipitation step) with greener alternatives such as deep eutectic solvents (DESs) could further improve the environmental sustainability of the extraction and preparation process and represents a promising direction for future optimization of HSSM‐CMC films (Castro‐Muñoz 2025). These investigations would contribute to a more complete evaluation of the suitability of HSSM‐CMC films as bio‐based active packaging materials for food applications.

## 6 Conclusion

The findings showed that HSSM can serve as a promising functional polymeric ingredient for producing biodegradable polymer films. Films were prepared using 1.25% HSSM and 1% CMC, with glycerol (10%, 15%, and 20% w/w) as a plasticizer. At 10% glycerol, films showed the lowest water vapor permeability (WVP, 1.35 × 10^−10^ g·s^−1^·m^−1^·Pa^−1^), elongation at break (EB, 17.67%), moisture content (18.33%), thickness (0.0235 mm), and WS (35.35%), while having the highest density (1.45 g/cm^3^) and TS (20.40 MPa). With increasing glycerol content, WVP and EB increased, whereas TS decreased. Furthermore, the film containing 1.25% HSSM and 10% glycerol showed antimicrobial activity against selected bacterial strains, with the strongest inhibition against *Bacillus cereus* and *Staphylococcus aureus* . The developed HSSM‐CMC films provide a renewable plant‐derived polymer matrix containing intrinsic bioactive compounds, with an optimized balance of mechanical, barrier, and functional properties. However, their hydrophilic nature and moisture sensitivity should be considered for practical applications.

Previous studies on plant mucilage‐based films have demonstrated biodegradation potential, such as complete soil dissolution of basil seed gum films within 60 days. Considering the similar glycerol‐dependent increases in hydrophilicity and WS observed in HSSM‐CMC films, these films may also possess biodegradation potential; however, direct experimental assessment is required to confirm this property.

Overall, HSSM‐based polymeric films represent a promising biodegradable polymeric matrix with an optimized balance of mechanical, barrier, and functional properties suitable for incorporation of active agents in advanced polymer applications.

## Author Contributions

**Maryam Beigomi:** conceptualization, investigation, writing – original draft, writing – review and editing, data curation, supervision, validation, methodology, project administration, visualization. **Mohammad Amin Mashhadi:** investigation, data curation. **Moharram Valizadeh:** software, formal analysis, resources. **Farzaneh Farajian Mashhadi:** writing – review and editing, data curation.

## Funding

The authors have nothing to report.

## Conflicts of Interest

The authors declare no conflicts of interest.

## Data Availability

The data that support the findings of this study are available from the corresponding author upon reasonable request.
